# Human pressures and ecological status of European rivers

**DOI:** 10.1038/s41598-017-00324-3

**Published:** 2017-03-16

**Authors:** B. Grizzetti, A. Pistocchi, C. Liquete, A. Udias, F. Bouraoui, W. van de Bund

**Affiliations:** European Commission, Joint Research Centre (JRC), Directorate D—Sustainable Resources, via Enrico Fermi 2749, 21027 Ispra, Italy

## Abstract

Humans have increased the discharge of pollution, altered water flow regime and modified the morphology of rivers. All these actions have resulted in multiple pressures on freshwater ecosystems, undermining their biodiversity and ecological functioning. The European Union has adopted an ambitious water policy to reduce pressures and achieve a good ecological status for all water bodies. However, assessing multiple pressures on aquatic ecosystems and understanding their combined impact on the ecological status is challenging, especially at the large scale, though crucial to the planning of effective policies. Here, for the first time, we quantify multiple human pressures and their relationship with the ecological status for all European rivers. We considered ecological data collected across Europe and pressures assessed by pan-European models, including pollution, hydrological and hydromorphological alterations. We estimated that in one third of EU’s territory rivers are in good ecological status. We found that better ecological status is associated with the presence of natural areas in floodplains, while urbanisation and nutrient pollution are important predictors of ecological degradation. We explored scenarios of improvement of rivers ecological status for Europe. Our results strengthen the need to halt urban land take, curb nitrogen pollution and maintain and restore nature along rivers.

## Introduction

In the second half of the 20^th^ century economic activities flourished in Europe while the status of rivers, lakes and coastal waters chronically deteriorated^1^. Human activities have produced multiple pressures on waters, including nutrient pollution^2, 3^, modifications of river morphology^4, 5^, alterations of water flow regime^6, 7^ and the introduction of alien species^8^. Multiple pressures from land-based activities pose threats to human water security and freshwater biodiversity^9^, and have produced cumulative effects in oceans and coastal waters^10^.

Natural spatio-temporal heterogeneity in rivers and floodplains is essential to support ecosystem biodiversity^11^. However river regulation, such as flow alterations, channelization, dredging and river bank stabilization, have reduced the connectivity in the riverine landscape and altered the fluvial dynamics that support habitat heterogeneity^11^. Similarly, the widespread construction of dams has diminished the natural disturbance patterns in rivers, homogenizing flow regional differences and creating cumulative transboundary effects^12, 13^. Freshwater biodiversity is further threatened by water pollution related to human activities in the catchment, fish overexploitation and the increase in the number of alien species^14^. All these actions have resulted in multiple pressures on freshwater ecosystems that undermine their biodiversity and ecological functioning.

Disentangling and quantifying the cause and effect relationship between multiple pressures and ecological functioning is challenging, especially when addressing large geographical areas like Europe. Firstly, the quantification of pressures on water systems is hampered by limited and spatially heterogeneous data. Secondly, multiple pressures are acting concurrently on water bodies and their combined effect is poorly understood^15^. Thirdly, ecological conditions are the result of impacts building up over time, local natural conditions and climatic variability^16, 17^. Finally, ecological systems could change following non-linear patterns and regime shifts, and restoration measures do not necessarily return the ecological systems to their original state^18^. All these aspects contribute to a great complexity in the link between multiple pressures and ecological status in water bodies. Yet understanding this relationship is necessary to plan effective policies^19, 20^ and restoration measures^21^, as long-term availability of water resources and many benefits for people depend on healthy aquatic ecosystems^22, 23^.

To protect and enhance water resources and aquatic ecosystems, since 2000 the European Union has adopted an ambitious water policy, the Water Framework Directive (WFD)^24^, with the objective of reducing pressures and achieving good ecological status for all European water bodies. With this aim, EU Member States had to assess the ecological status of rivers, lakes and coastal waters in their territory, and established programmes of measures to reduce significant anthropogenic pressures affecting the status.

Here, for the first time, we have characterised the main pressures acting on European rivers and explored their relationship with the ecological status reported by EU Member States. Our analysis addressed three main questions: (1) How do multiple pressures affect the ecological status of European rivers? (2) To what extent has the EU water policy target of good ecological status been achieved? and (3) How and where would measures to improve the ecological status of rivers be effective?

## Results

### How do multiple pressures affect the ecological status of European rivers?

To address this first question we quantified multiple pressures on European rivers and examined their relationship with reported data on the ecological status.

**Figure 1:**
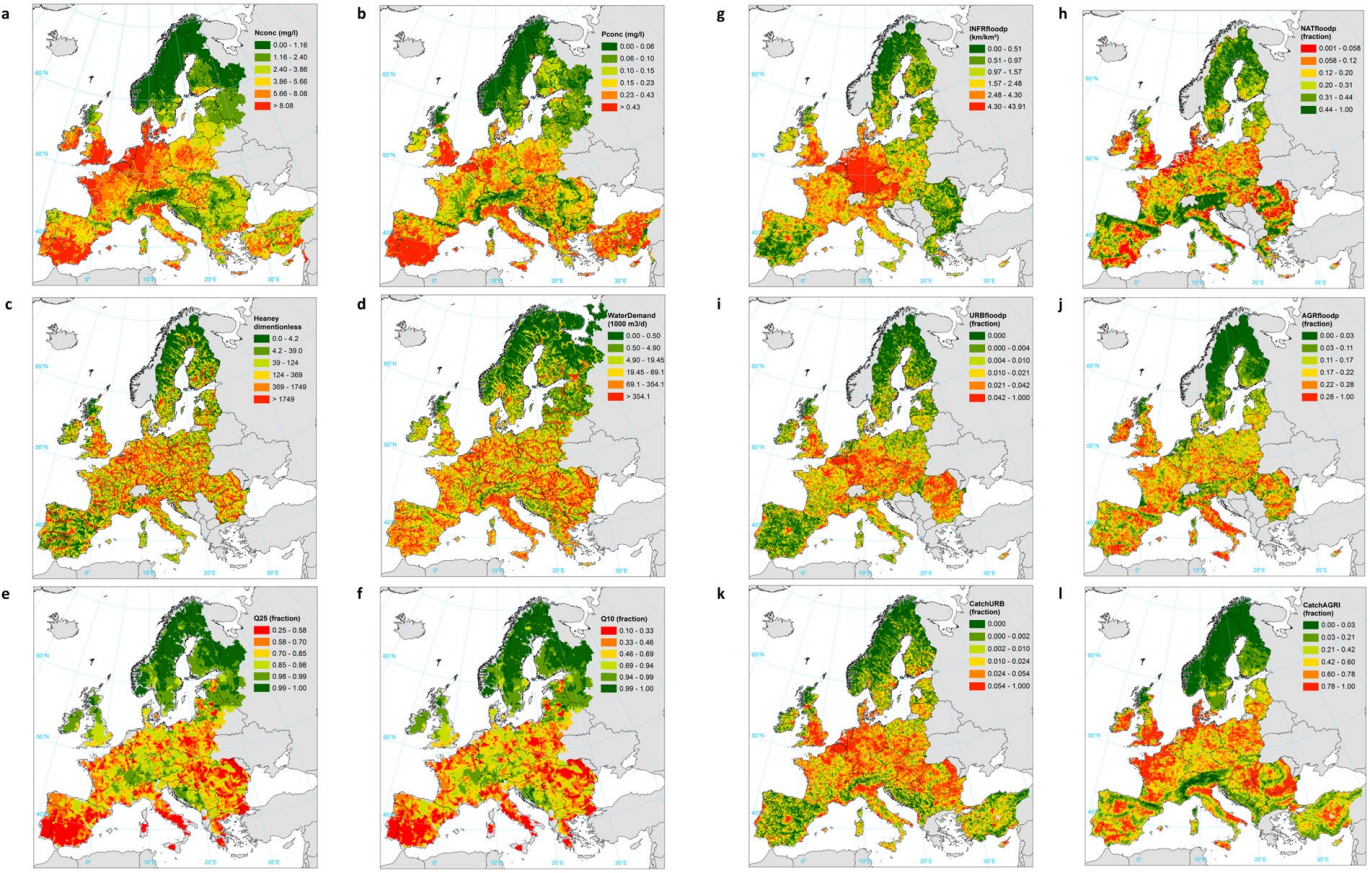
Maps of pressures on European rivers. (**a**) Nitrogen concentration; (**b)** phosphorus concentration; (**c**) pollution from urban runoff; (**d**) water demand; (**e**) preservation of low flow at 25^th^ percentile; (**f**) preservation of low flow at 10^th^ percentile; (**g**) infrastructures in floodplains; (**h**) natural areas in floodplains; (**i**) urban areas in floodplains; (**j**) agricultural areas in floodplains; (**k**) artificial land cover in catchment area; (**l**) agricultural land cover in catchment area. Details of the pressures indicators are in Table 1. Maps generated with ArcGIS 10.1 for desktop (http://www.esri.com/software/arcgis).

According to a recent European Commission report^25^, the major pressures acting on European rivers are related to pollution, hydrological changes and hydromorphological alterations. We considered 12 indicators that could inform on these pressures (Table 1): nitrogen and phosphorus concentration; pollution from urban runoff; water demand; alteration of natural low flow regimes (at 10^th^ and 25^th^ percentiles); density of infrastructure in floodplains; natural areas in floodplains; artificial and agricultural land cover in floodplains; and artificial and agricultural land cover in the drained area. We quantified these indicators at the spatial resolution of catchments (180 km^2^ on average), using pan-European models and data sets (we used best available data for the period 2004–2009, see ‘Methods’). The maps of pressures on European inland waters are shown in Fig. 1.

**Table 1:** Pressures considered in the study and the respective indicators.

Pressure	Indicator (*acronym*)	How the indicator is estimated (reference year and available spatial coverage*)
Pollution	Nitrogen concentrations in rivers (*Nconc*)	Estimated nitrogen concentration in rivers (mgN/l), based on the model GREEN^35^. (2005; EU-28+)
	Phosphorus concentrations in rivers (*Pconc*)	Estimated phosphorus concentration in rivers (mgP/l), based on the model GREEN^35^. (2005; EU-28+)
	Diffuse pollution from urban runoff (*Heaney*)	Relative intensity of the potential pollution load from urban runoff (dimensionless), estimated by the Heaney model^34, 36^. The indicator is designed to reproduce potential pollution and not specific contaminants, based on urban land cover (CLC 2006), annual precipitation and population. (2006; EU-28, without GR and CY)
Hydrological alterations	Total water demand (*WatDemand*)	Total water demand in the catchment upstream (mm/day) (ref. 34 based on ref. 37). (2006; EU-28, without CY)
	Low flow alteration at 25%-ile (*Q25*)	Ratio between the number of days the water flow is below the 25%-ile with and without water abstractions (fraction)^34^. The flow duration curve without abstractions is used to define the flow threshold of Q25%-ile. The indicator is computed using the estimations of the hydrological model LISFLOOD^37^, considering baseline conditions including water abstractions and an ideal undisturbed case with no abstractions. (2006; EU-28, without CY)
	Low flow alteration at 10%-ile (*Q10*)	Ratio between the number of days the water flow is below the 10%-ile with and without water abstractions (fraction)^34^. The flow duration curve without abstractions is used to define the flow threshold of Q10%-ile. The indicator is computed using the estimations of the hydrological model LISFLOOD^37^, considering baseline conditions including water abstractions and an ideal undisturbed case with no abstractions. (2006; EU-28, without CY)
Hydro-morphological alterations	Density of infrastructures in floodplains (*INFRfloodp*)	Density of infrastructure (roads and railways) in the floodplains (km/km^2^)^34, 40^. (dates not available, data extracted in 2014; EU-28, without HR)
	Natural areas in floodplains (*NATfloodp*)	Fraction of the floodplain occupied by natural elements^30, 38^. (2000; EU-28, without HR)
	Artificial land cover in floodplains (*URBfloodp*)	Fraction of urban land use (CLC 2006 class: artificial areas) in the floodplains^34^. (2006; EU-28, without GR and HR)
	Agricultural land cover in floodplains (*AGRfloodp*)	Fraction of agricultural land use (CLC 2006 class: arable land and permanent crops) in the floodplains^34^. (2006; EU-28, without GR and HR)
Integrated	Artificial land cover in catchment area (*catchURB*)	Fraction of catchment area which is urban (CLC 2006 class: artificial areas)^34^. (2006; EU-28, without GR and HR)
	Agricultural land cover in catchment area (*catchAGRI*)	Fraction of catchment area which is agricultural (CLC 2006 class: arable land and permanent crops)^34^. (2006; EU-28, without GR and HR)

(*) As at January 2017 the European Union (EU) is composed of 28 Member States (MS): Belgium (BE), Bulgaria (BG), Czech Republic (CZ), Denmark (DK), Germany (DE), Estonia (EE), Ireland (IE), Greece (GR), Spain (ES), France (FR), Croatia (HR), Italy (IT), Cyprus (CY), Latvia (LV), Lithuania (LT), Luxembourg (LU), Hungary (HU), Malta (MT), Netherlands (NL), Austria (AU), Poland (PO), Portugal (PT), Romania (RO), Slovenia (SI), Slovakia (SK), Finland (FI), Sweden (SE) and United Kingdom (GB).

In parallel, we computed a proxy indicator of the ecological status of rivers (at the same spatial resolution of pressures), based on the data reported by EU Member States (Supplementary Information Figure S1). The ecological status is an integrative evaluation of aquatic ecosystem health, designed to reflect changes in community structure and ecosystem functioning in response to anthropogenic pressures^26^. It is expressed in five classes—high, good, moderate, poor and bad—and its assessment is carried out by EU Member States (per single water body), using biological assessment methods. The national classification scales are harmonised by intercalibration to assure their consistency at the EU level. The target set by EU water policy is to reach a good ecological status for all rivers (by 2015 or 2027). Our proxy indicator for the ecological status of European rivers covers 77% of the EU’s surface. Out of this area, 38% is estimated to be in good or high ecological status, 42% in a moderate state and the rest in poor or bad status.

**Figure 2:**
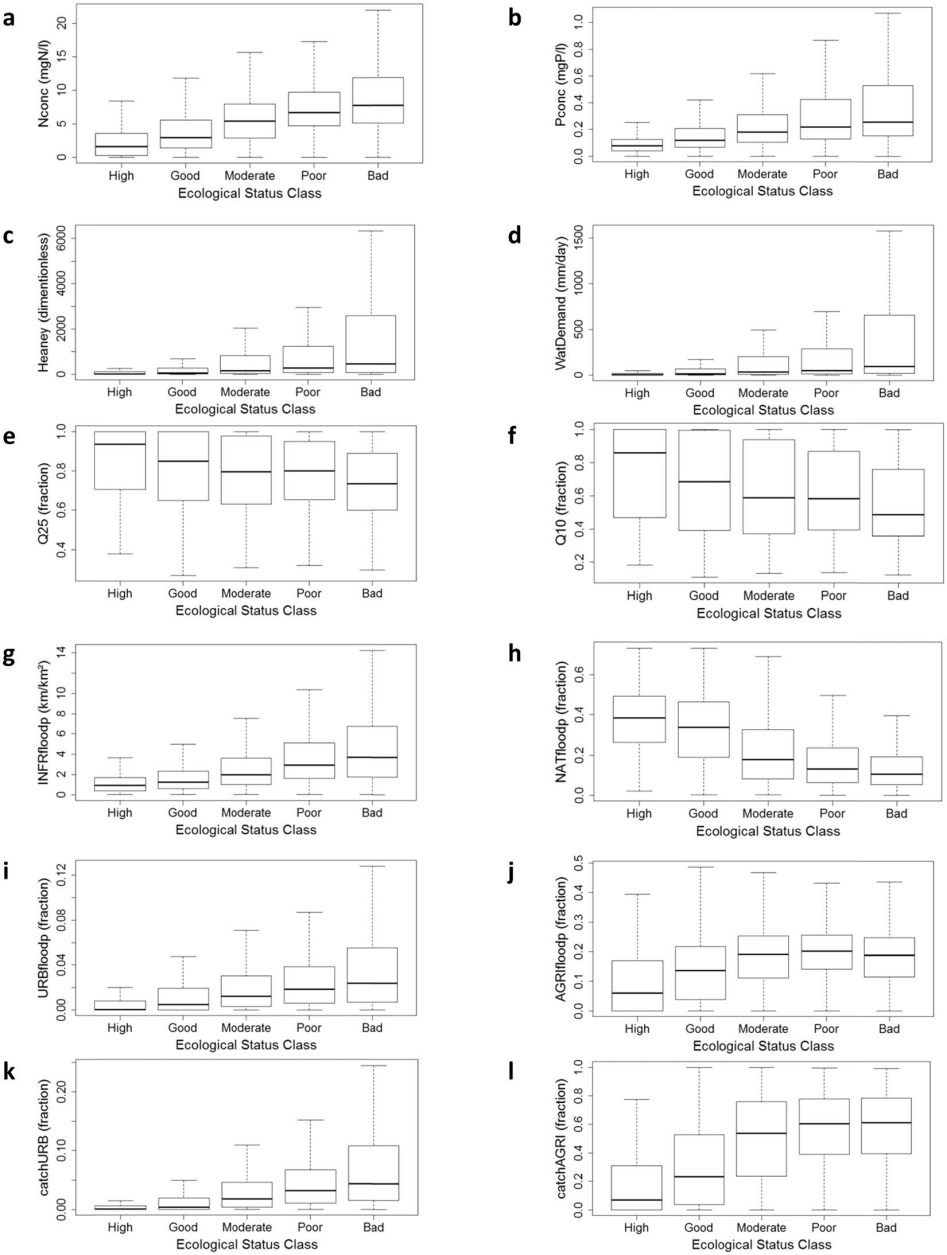
Relationship between the indicators of pressures and the proxy of the ecological status. (**a**) Nitrogen concentration; (**b**) phosphorus concentration; (**c**) pollution from urban runoff; (**d**) water demand; (**e**) preservation of low flow (at 25^th^ percentile); (**f**) preservation of low flow (at 10^th^ percentile); (**g**) infrastructures in floodplains; (**h**) natural areas in floodplains; (**i**) urban areas in floodplains; (**j**) agricultural areas in floodplains; (**k**) artificial land cover in catchment area; (**l**) agricultural land cover in catchment area. The indicators of pressures are described in Table 1.

When looking at the distribution of individual pressures per class of ecological status, we observe significant correlations and trends in the expected direction (Fig. 2). For all indicators of pressures medians significantly differ per class of ecological status (Kruskall–Wallis test, p < 0.05). Nitrogen and phosphorus concentrations increase towards poor and bad ecological classes, and the same happens for the indicators of hydromorphological alterations in floodplains. Also, pressures related to urban and agricultural land in the drained area take higher values in poor and bad classes, while greater maintenance of natural low flow and the presence of natural riparian areas are related to good and high ecological status.

**Figure 3:**
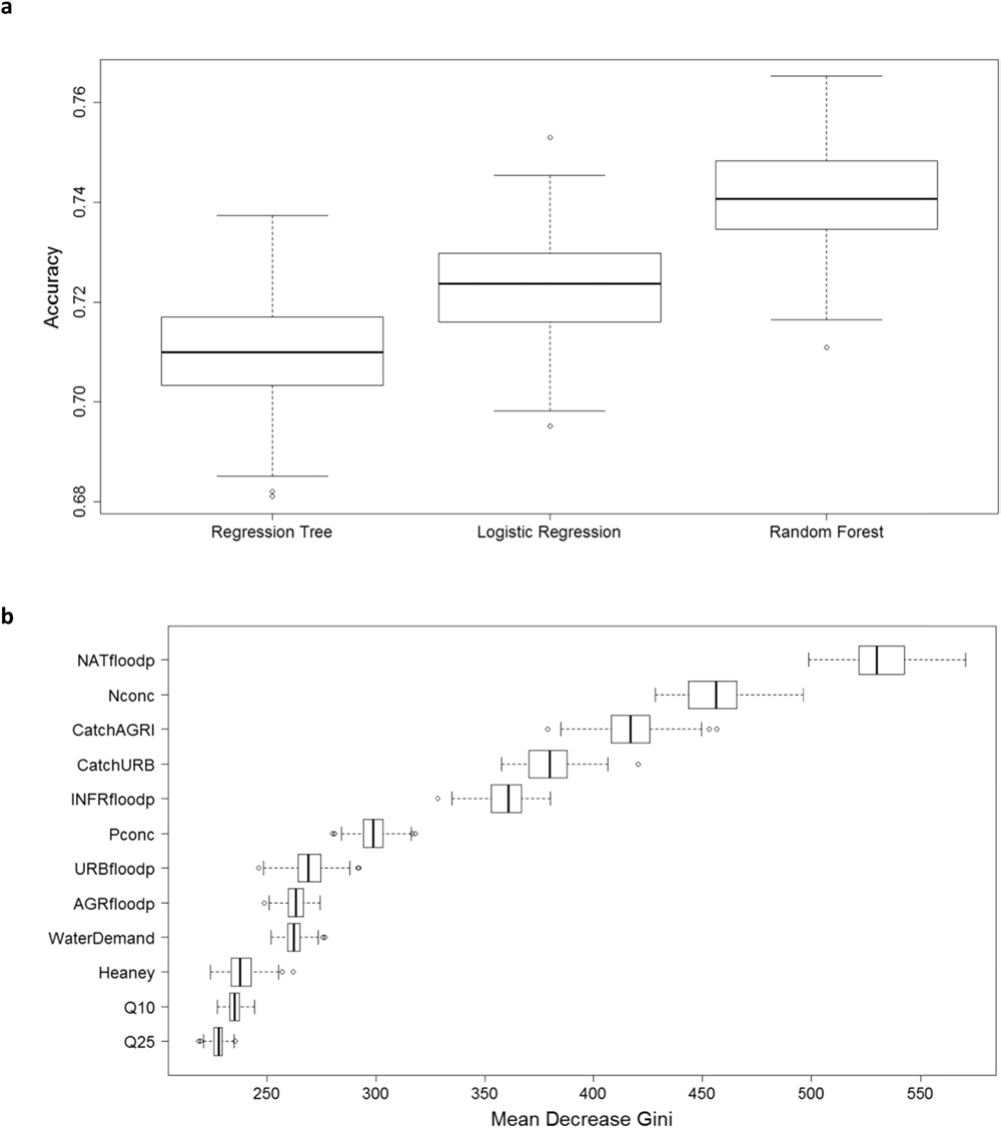
Model results. (**a**) Accuracy of classification using the regression tree (RT), logistic regression (LR) and random forest (RF) models. (**b**) Importance of the variables in the classification of the random forest method computed by the mean decrease Gini index^48, 49^. The analysis refers to the period 2004–2009, for which data on the ecological status were reported and most of the pressures indicators were available.

We explored the combined effects of multiple pressures on the achievement of good ecological status of rivers, applying statistical classification methods (notably, regression tree (RT), logistic regression (LR) and random forest (RF)). The accuracy of the models’ predictions was up to 0.74 (0.70 for RT, 0.72 for LR and 0.74 for RF respectively, Fig. 3a). The results of the models showed that the good ecological status of rivers is explained by a combination of pressures, and the most important predictors are the presence of natural areas in floodplains, nutrient concentration (especially nitrogen), infrastructures in floodplains and urbanisation and agriculture in the drained catchment (Fig. 3b).

### To what extent has the EU water policy target of good ecological status been achieved?

**Figure 4:**
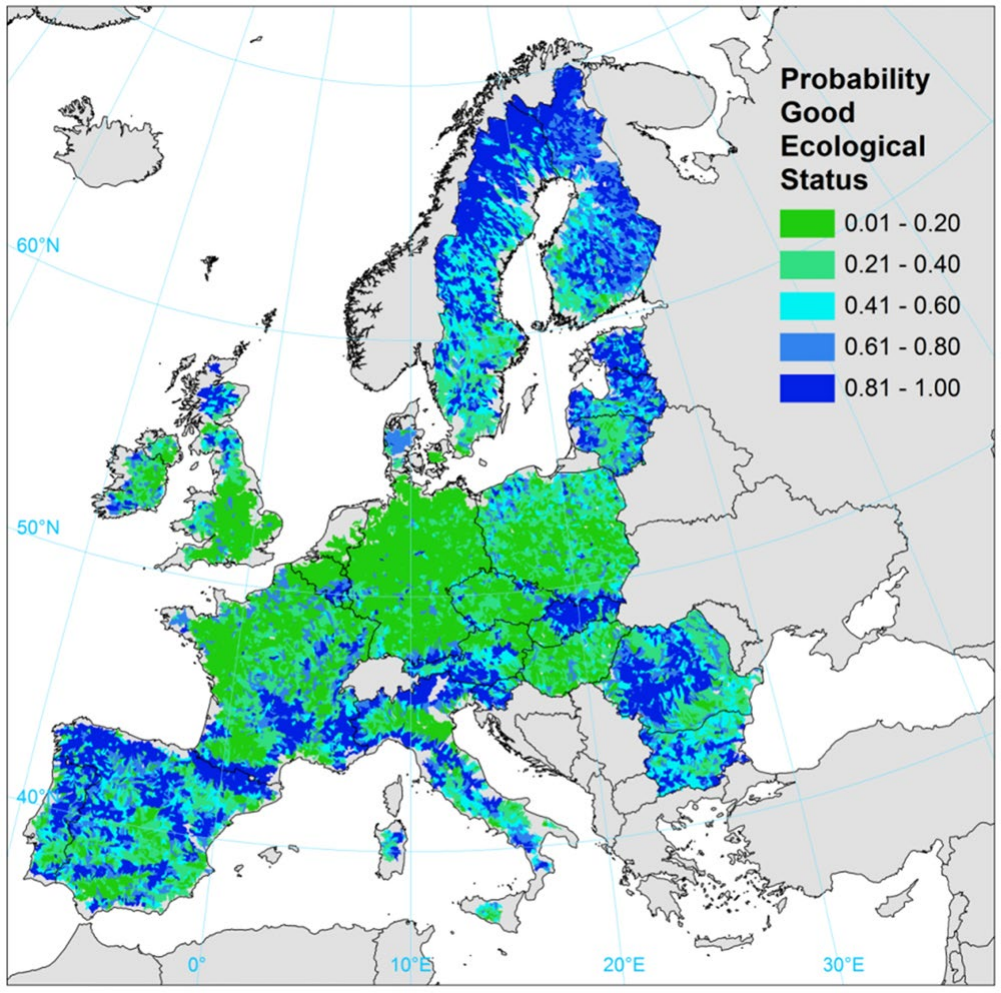
Probability of good ecological status of rivers. Values estimated by the random forest method applied to all catchments with complete data on pressures (89% of EU). Map generated with ArcGIS 10.1 for desktop (http://www.esri.com/software/arcgis).

To examine this second question, we estimated the level of achievement of the EU water policy objective, using the relationship established by modelling (RF). We estimated the probability of meeting the policy target of good ecological status for all EU rivers in catchments with complete data on pressures (89% of the EU’s surface), therefore, also in areas where direct measurements of ecological status were not available. According to our estimations, the proportion of the EU surface where rivers meet the water policy target, with a probability of at least 70%, is 32% (Fig. 4).

**Figure 5:**
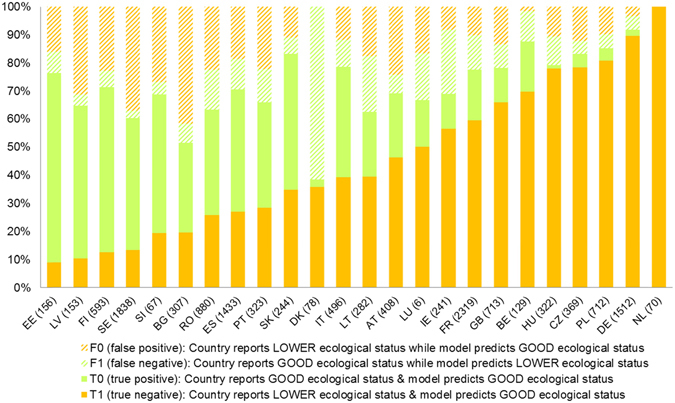
Distribution of model accuracy and errors per country. The values within brackets indicate the number of catchments with available data. Results are based on the random forest method. The analysis refers to the period 2004–2009, for which data on the ecological status were reported and most of the pressures indicators were available.

The distribution of the model’s accuracy and error type per country can provide more insights (Fig. 5). False negatives (9%, the country reports meeting the target while the model predicts not meeting the target) could indicate where pressures are overestimated by the European assessment or local measures are not taken into account. For example, this could be the case of Denmark, where substantial investments have been made in the restoration of wetlands^27^. On the other hand, false positives (17%, the country reports not meeting the target while the model predicts meeting the target) could suggest where pressures are underestimates or not captured by the current indicators. This could be the case of Sweden, where local water flow modifications could be the reason for not achieving the good ecological status^28^. Among errors, dominance of false positives could characterise countries that adopt stricter rules or more conservative reference status in the implementation of the WFD, compared to the average of EU countries. Contrarily, dominance of false negatives might occur for countries that have slightly lower standards or consider a partially impacted ecological status as reference conditions for the water bodies.

Besides misrepresentation of pressures and local measures, or difference in reference status among the national assessments, another reason that could explain the model errors is a different interaction of multiple pressures according to river typology or ecological regions. However, overall, discrepancies between model predictions and the ecological status reported by the countries are spread homogeneously across the study area, indicating no particular bias in the assessments by Member States. This is an encouraging signal considering the large effort spent on the national assessments and on the intercalibration of methods among Member States.

### How and where would measures to improve the ecological status of rivers be effective?

**Figure 6:**
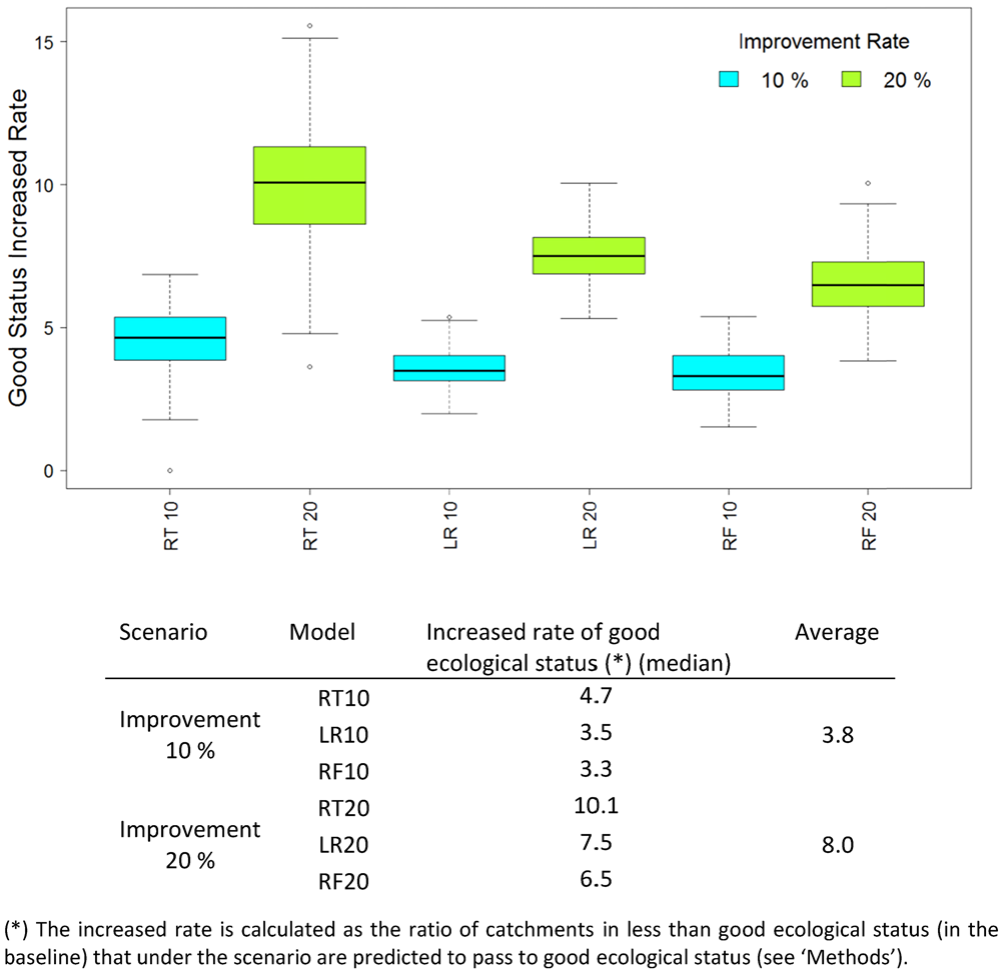
Scenarios of measures for improvement of river ecological status. The scenarios are simulated by the three classification methods: regression tree (RT), logistic regression (LR) and random forest (RF). The scenarios ‘measures for improvement’ estimate the effects of contemporary reduction of nitrogen concentration in rivers and the increase of natural areas in floodplains, considering improvement rates of 10% and 20%.

To shed light on this third question, we examined the effects of measures to improve the ecological status of rivers through scenario analysis. We tested the scenario of concurrently reducing nitrogen pollution and increasing natural areas in floodplains (using RT, LR and RF models, Fig. 6), as these pressures were among the most significant variables explaining the good ecological status (according to the results of the RF, Fig. 3). The analysis showed that 4% of EU catchments with degraded rivers would achieve a good ecological status by reducing nitrogen pollution and increasing natural areas in floodplains by 10%, and up to 8% of catchments could meet the policy target if the same measures were raised to 20%. However, this is a conservative estimation, as the methods adopted do not include the effect of improving the ecological quality in one catchment on the downstream area.

Yet the scenario analysis helps us understand how addressing a combination of pressures can affect the ecological status compared to changes in single pressures (which are presented in Supplementary Information Figures S2, S3 and S4) and where measures are likely to yield good ecological status. According to our results, the predicted increase in good ecological status by simultaneously reducing nitrogen concentration in rivers and enhancing natural areas in floodplains is slightly higher than the sum of the predicted increase by changing the two pressures independently, showing a synergistic effect.

## Discussion

Statistical classification models, as adopted here, cannot bring strong evidence of a causal relationship between the pressures and the ecological status, but they can unveil patterns. Our results show that the ecological status of European rivers can be explained by multiple pressures, and in particular by a combination of local pressures (i.e. hydromorphological alterations) and catchment pressures (i.e. nutrient pollution and land use). Measures to improve the ecological quality of rivers should consider these two dimensions, as well as synergistic effects of acting simultaneously on more pressures.

In our analysis, flow regime alteration and water abstractions appeared less significant. They were probably not completely represented by selected indicators or spatial information. At the same time, it is currently under debate whether the present assessment of the ecological status sufficiently accounts for hydrological alterations of river ecosystems^29^. Other pressures not included in this study might also be relevant to explaining the ecological status, such as the disruption of upstream-downstream connectivity, historical impacts having legacy effects and the introduction of invasive species. In addition, the river typology could explain the different impact of similar pressures combination.

The joint effort of EU Member States in monitoring the ecological status remains crucial to ensuring that effective measures for protecting and restoring aquatic ecosystems are deployed, considering the panoply of vital ecosystem services they provide^30, 31^. Similarly, models and remote sensing data represent useful tools to assess multiple pressures across Europe, especially in less data intensive areas.

Our results indicate that maintaining natural floodplains and limiting nitrogen pollution can be key measures to improve the ecological status of rivers and achieve water policy goals, producing synergetic effects. They also suggest that preserving natural land cover as opposed to urban sprawling, which erodes the capacity of the ecosystem to buffer pressures^32^, should be seen an investment in ecosystem resilience. Overall, our results confirm evidence of the need to halt urban land take, curb nutrient pollution and preserve natural areas along water courses, in order to protect the ecological quality of rivers and ensure future benefits for humans.

## Methods

### Spatial extent and resolution

The area covered by the study is the European Union (EU). As at January 2017 the EU is composed of 28 Member States (notably Belgium, Bulgaria, Czech Republic, Denmark, Germany, Estonia, Ireland, Greece, Spain, France, Croatia, Italy, Cyprus, Latvia, Lithuania, Luxembourg, Hungary, Malta, Netherlands, Austria, Poland, Portugal, Romania, Slovenia, Slovakia, Finland, Sweden, United Kingdom). We based the spatial analysis on a consistent hydrological geodatabase covering Europe, with elementary catchments of 180 km^2^ on average, called the HydroEurope database^33^. For inland waters the EU is divided into 23 187 catchments, corresponding to 4 098 757 km^2^. In the study, we referred to this area as reference for the EU, although it is slightly less (7%) than the EU surface, as small coastal catchments are not included in the database.

### Multiple pressures

The anthropogenic pressures on aquatic ecosystems were identified based on the Common Implementation Strategy (CIS) and the first River Basin Management Plans (RBMPs) submitted by the EU Member States^25, 34^. The main types of pressures reported for river ecosystems were nutrient and chemical pollution, hydrological alterations and morphological modifications. We proposed a set of 12 indicators that could inform on the quantitative presence of these pressures and could be computed consistently across Europe, using already established models or available spatial data, considering the best available data for the period 2004–2009. The indicators of pressures proposed in this study are summarised in Table 1, including the available reference year and the spatial coverage. For pollution, nitrogen and phosphorus concentration in surface waters were considered, based on the nutrient loads estimated by the GREEN model combined with water flow estimated by a simple hydrological model based on a Budyko framework^35^. In addition, load from urban runoff was estimated by an indicator accounting for urban population and rainfall, derived from the loading function proposed by Heaney *et al.* ^36^, as described in Pistocchi *et al.* ^34^. For hydrological alteration, the total water demand was derived from the European maps at 5 km resolution used as input by the LISFLOOD hydrological model^37^. These include water demand for irrigation, public supply, industry (including energy production) and livestock. The indicators of flow regime alteration were computed as the number of days in which the actual stream flow is below the 10^th^ and 25^th^ natural flow percentile, normalised by the corresponding natural duration (i.e. 36.5 and 91.25 days respectively). The actual and natural flow duration curves were estimated using the LISFLOOD model under the 2006 baseline conditions, in presence and in absence of water abstractions respectively^34, 37^. A series of (proxy) indicators of hydromorphological pressures were considered to reflect the conditions of floodplains, including the share of the floodplain occupied by agricultural land, by artificial areas and by natural areas (riparian functional areas), and the density of infrastructures (roads and railways) in the floodplain. Floodplains were identified through the data set described by Clerici *et al.* ^38^. Agricultural and artificial land cover shares were estimated on the basis of the CORINE Land Cover 2006 map^39^. Infrastructures were extracted from the freely accessible OpenStreetMap data set^40^. The presence of riparian functional areas was calculated as the average riparian vegetation buffer width divided by the floodplain width, where the average riparian vegetation buffer width was derived by aggregation of the vegetation maps developed by Weissteiner *et al.* ^41^. All variables relating to floodplains were aggregated at 1 km resolution across the stream network. Finally, the fraction of the drained catchment occupied by urban areas and by agricultural land were considered as two additional integrated indicators of pressures on rivers related to the land use in the catchment. All pressures indicators were computed or aggregated at the spatial resolution of catchments of the HydroEurope database^33^ (Fig. 1).

### Ecological status

The ecological status is a synthetic judgement that represents the condition of water bodies as high, good, moderate, poor or bad, based on assessment methods for biological quality elements (BQEs, that are phytoplankton, flora, invertebrate fauna and fish fauna), combined with information on physico-chemical and hydromorphological conditions. The ecological status is defined in general terms by the WFD, which is the EU water law; then each individual Member State develops national assessment methods. Depending on the Member State, the assessment of the ecological status was based on full BQEs, pressure assessments, expert judgement or combinations of the above. This variability in approaches limits the methodological consistency across the EU. However, classification scales for the biological classification methods have been intercalibrated across EU Member States^42–44^.

For this study, we used ecological status data from River Basin Management Plans reported according to Article 13 of the WFD, extracted from the WISE2 database, compiled by the European Environment Agency^45^, including data from 2004 to 2009. For each monitored river stretch the data set reports the class of ecological status or potential and the length of the stretch. A river stretch is defined as a water body in the WFD. Only the coordinates of the centroid of each water body were available for this study, while the geographic delineation of the stretch was not available at the European scale. To overcome this lack of information and the different spatial density of monitoring across the EU, we developed a proxy indicator of the ecological status of rivers that could be representative at the scale of HydroEurope catchments, the same spatial unit at which pressure indicators were aggregated. For each catchment, we considered the ecological status assigned to all centroids of water bodies falling in that catchment, yielding valid and usable data for 79 630 water bodies across the EU. Then, for each catchment, we computed the percentage of monitored river length under each class of ecological status (with 1 = High, 2 = Good, 3 = Moderate, 4 = Poor, 5 = Bad) and the dominant class CMODE (corresponding to the mode), i.e. the class that appears most often in the total monitored length of the observations. CMODE takes values between 1 and 5, corresponding to the five classes of ecological status (Supplementary Information Figure S1). We also considered a simple Boolean variable called TARGET to indicate if the good ecological status is met or not. TARGET takes value 0 when the sum of percentages of monitored river length in high and good ecological status is higher than the sum of percentages in moderate, poor and bad status, and takes value 1 otherwise. Therefore, TARGET is a proxy indicator of meeting the WFD target of good ecological status.

### Data sample

The spatial extent covered by the 12 pressures indicators varies depending on the input data used in each pressure assessment (see the specific extent covered in Table 1). We did not have complete information on pressures for four countries—Greece, Croatia, Cyprus and Malta—whose surface represents about 5% of the EU. We could develop a completed data set of pressures for 89% of the EU’s surface (85% of catchments). Data on rivers’ ecological status were available for 15 052 catchments of HydroEurope (65% of EU catchments, 77% of the EU’s surface). In total, there were 13 651 catchments with complete indicators of pressures and complete data on ecological status that we used for the models’ calibration. This represents 59% of the catchments and 71% of the EU’s surface. The temporal extent of the analysis refers to the period 2004–2009, for which data on the ecological status were reported and most of the pressures indicators were available.

### Analysis

We explored the data distribution and correlation, and we performed a factor analysis. We analysed the distribution of values of each indicator of pressures per class of ecological status, using the most frequent status class reported per catchment CMODE as proxy for the ecological status. We assessed for all indicators of pressures that the medians per class of ecological status were significantly different (p < 0.05) by a Kruskall–Wallis test (Fig. 2).

We applied statistical methods to investigate how multiple pressures can explain the ecological status in rivers, using the variable TARGET as indicator of meeting the policy objective in each catchment. Specifically, we considered three types of classification techniques: regression trees^46^ (RT), logistic regression^47^ (LR) and random forest^48^ (RF). These methods establish a classification of catchments using the information embedded in the data. We applied the three methods using the complete data set on pressures and ecological status. This means that the temporal extent of the analysis does not refer to a specific year but is centred on the period 2004–2009.

For the analysis the three classification methods (RT, LR and RF) were applied 200 times using random samples (without replacement) extracted from the data set. Each iteration included three steps: 1. randomly balance the data set (as the number of catchments with TARGET = 1 exceeded those with TARGET = 0); 2. randomly select, out of the balanced data set, a training sample (80% of data) and a testing sample (the 20% remaining); and 3. run the three models (RT, LR and RF) using the training sample (model calibration). Then the accuracy of the models was measured using the testing sample (model verification), as the ratio of samples (catchments) whose value (TARGET) is correctly predicted over the total number of samples (Fig. 3). The overall accuracy of each method was reported as the median of the 200 model runs.

The RT and RF models were set including all 12 pressure indicators as explanatory variables. The LR model was run firstly with 12 pressures and then including only the significant variables (p < 0.1 two-sided) and with sign coherent with the expected physical effect on ecological status. The importance of the variables in the classification of the random forest method was computed by the mean decrease Gini index^48, 49^.

We used the RF method (and the variable TARGET) to predict the probability of meeting the policy target of good ecological status in all EU catchments for which we had complete pressures indicators (89% of the EU’s surface) (Fig. 4). For reporting the EU’s area meeting the policy target we considered a probability threshold of 0.7.

Similarly, we based the analysis of predictions’ accuracy and errors per EU country on the RF method (Fig. 5), showing where modelled and reported ecological status were in agreement on meeting (T0) or non-meeting (T1) the policy target of good ecological status, and the frequency of false positive (F0, the model predicts meeting the target while the reported data indicate lower ecological status) and false negative (F1, the model predicts not meeting the target while the reported data indicate at least good ecological status).

Finally, we simulated two types of scenarios: the effect of measures for improvement of the ecological status (Fig. 6) and the effect of further degradation (Supplementary Information Figure S5), using the three methods, RT, LR and RF (and the variable TARGET). In the scenario ‘measures for improvement’ we tested the concurrent reduction of nitrogen concentration in rivers (−10% and −20%) and increase of natural areas in floodplains (+10% and +20%), while in the scenario ‘further degradation’ we simulated the simultaneous increase of nitrogen concentration in rivers (+10% and +20%) and reduction of natural areas in floodplains (−10% and −20%). The effects of the changes were quantified as the increase rate of catchments predicted in good ecological status (meeting the target of the water policy) compared to the baseline. For the scenarios, the models were run according to the three-step iteration presented above, and the effects tested on the catchments correctly classified by the models. We reported the overall expected effect of the scenarios as the average of the medians of the three models’ predictions. We also simulated a variation of ±10% and ±20% of one pressure at a time, using the three methods (RT, LR and RF). The results are shown in the Supplementary Information Figures S2, S3 and S4.

## Electronic supplementary material

Supplementary Information
