# Erratum: Human pressures and ecological status of European rivers

**DOI:** 10.1038/s41598-017-04857-5

**Published:** 2017-07-26

**Authors:** B. Grizzetti, A. Pistocchi, C. Liquete, A. Udias, F. Bouraoui, W. van de Bund

**Affiliations:** European Commission, Joint Research Centre (JRC), Directorate D—Sustainable Resources, via Enrico Fermi 2749, 21027 Ispra, Italy

*Scientific Reports* **7**:205; doi:10.1038/s41598-017-00324-3; Article published online 16 March 2017

This Article contains an error in Figure 5, where ‘T1 (true negative)’ should read:

“Country reports LOWER ecological status & model predicts LOWER ecological status”.

The correct Figure 5 appears below as Figure 1.

**Figure 1:**
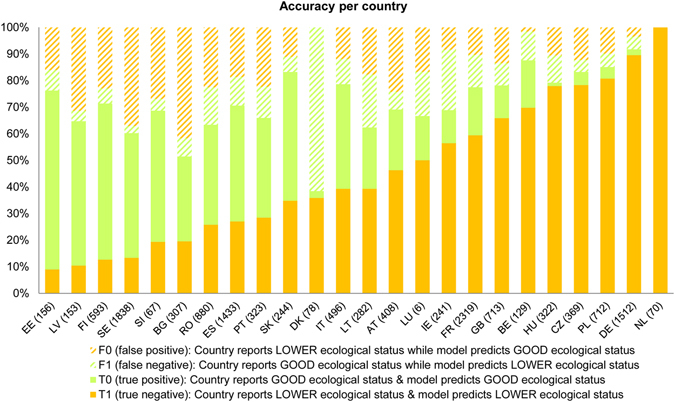
Distribution of model accuracy and errors per country. The values within brackets indicate the number of catchments with available data. Results are based on the random forest method. The analysis refers to the period 2004–2009, for which data on the ecological status were reported and most of the pressures indicators were available.

