# Supplementary material for: Human pressures and ecological status of European rivers
Source: Sci Rep. 2017 Mar 16;7:205. doi: 10.1038/s41598-017-00324-3 (PMC5428267; doi:10.1038/s41598-017-00324-3)
Supplement: Supplementary file 1 — Supplementary Information [file 41598_2017_324_MOESM1_ESM.pdf]

Supplementary Information

Article: "Human pressures and ecological status of European rivers"

Grizzetti, B., Pistocchi, A., Liquete, C., Udias, A., Bouraoui, F., van de Bund, W.

Reporting results

In the study, we expressed the results as fraction of EU area or as fraction of number of catchments in the HydroEurope database, since this was the spatial unit for the assessment of indicators of pressures and ecological status. However, considering the relatively homogeneous size of the catchments in the geodatabase, the latter are quite consistent with values reported by areas.

In the light of these considerations, the ratio of rivers in good ecological status according to our proxy of ecological status (which covers 77% of the EU's surface, Supplementary Information Figure S1) can be computed as fraction of catchments (the number of catchments in good ecological status, or TARGET=0, out of the total sample), fraction of area (area of catchments in good ecological status out of the total area of catchments with measurements) or fraction of river length (length of river stretches in good ecological status out of the total length for which information is reported). (Following these three approaches the ratio of rivers in good ecological status according to our proxy indicator for ecological status is estimated to be 36%, 38% and 32% respectively.) However, we could not always express the results by river length as this information was only reported in the data of ecological status by the Member States and was incomplete (information was not available outside the measured river stretches and did not refer to a common consistent river network for Europe). For this reason, the estimation of the probability of meeting the policy target of good ecological status (which covers 89% of the EU's surface and is based on model results, Figure 4) could only be expressed as fraction of area (32%) or catchments (28%).

Supplementary Information Figure S1 | Proxy of ecological status. Classes indicate the dominant ecological status class of measurements for rivers reported by Member States within the catchment (average size of the catchments is 180 km^2). The analysis refers to the period 2004-2009, for which data on the ecological status were reported. Map generated with ArcGIS 10.1 for desktop (http://www.esri.com/software/arcgis).

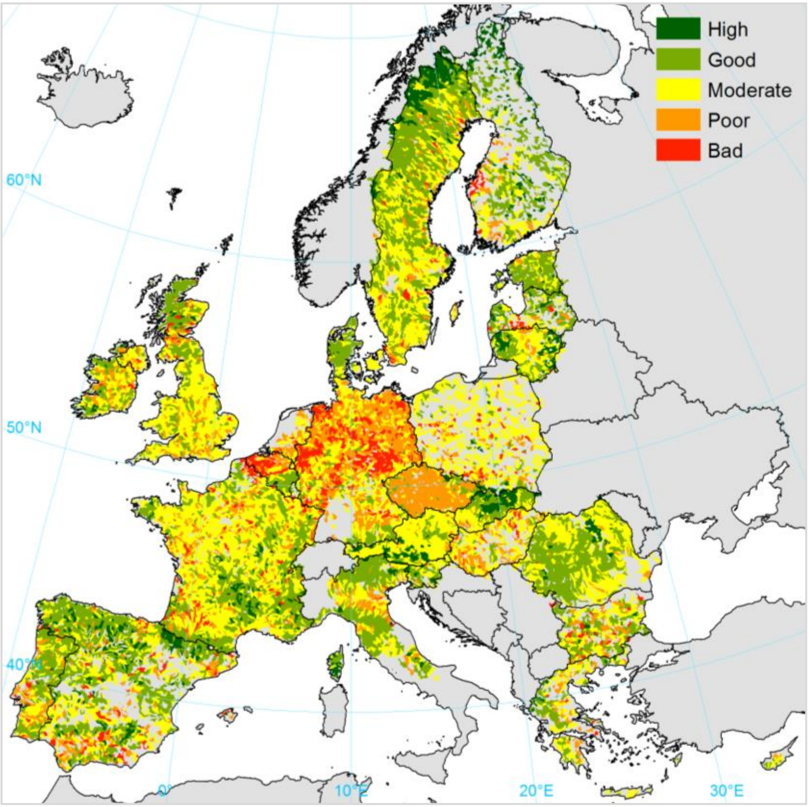

Supplementary Information Figure S2 | Expected effect of changing one pressure at a time on meeting the good ecological status, simulated by the regression tree (RT) method. a. changing single variable by ± 10%. **b**. changing single variable by ± 20%. The good status increased rate is calculated as the ratio of catchments in less than good ecological status (in the baseline) that under the scenario are predicted to pass to good ecological status.

a

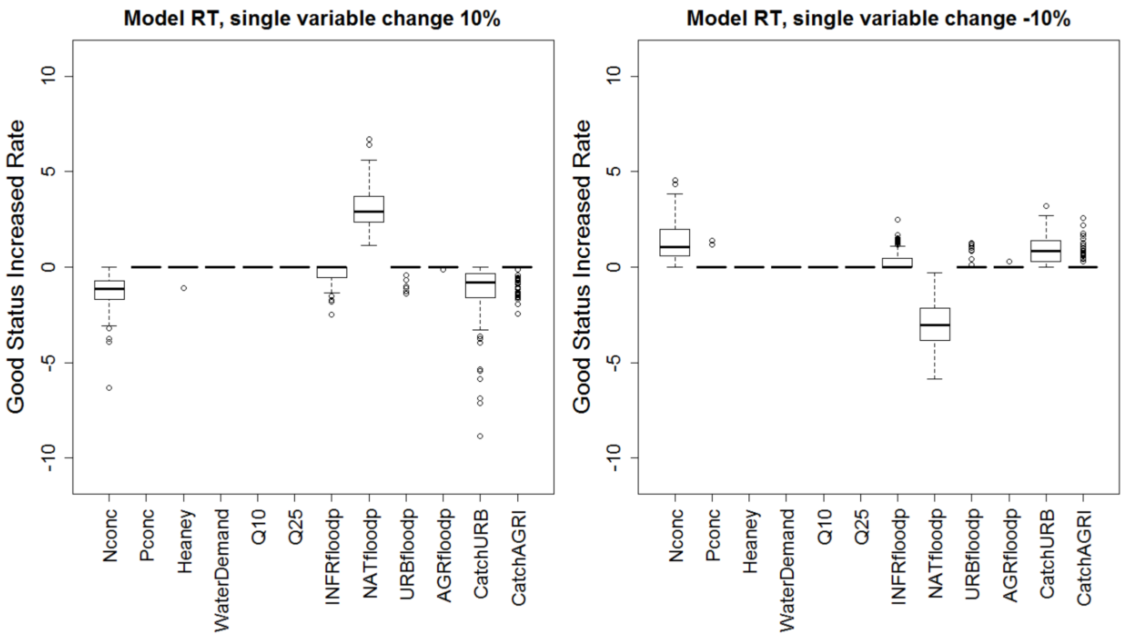

b

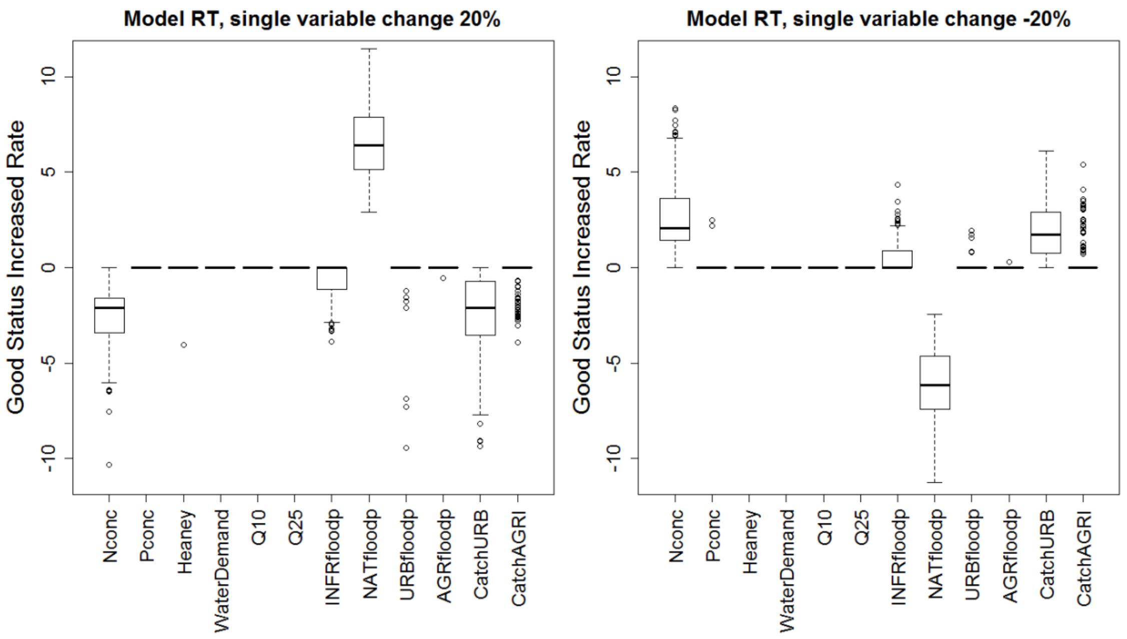

Supplementary Information Figure S3 | Expected effect of changing one pressure at a time on meeting the good ecological status, simulated by the random forest (RF) method. a. changing single variable by ± 10%. **b**. changing single variable by ± 20%. The good status increased rate is calculated as the ratio of catchments in less than good ecological status (in the baseline) that under the scenario are predicted to pass to good ecological status.

a

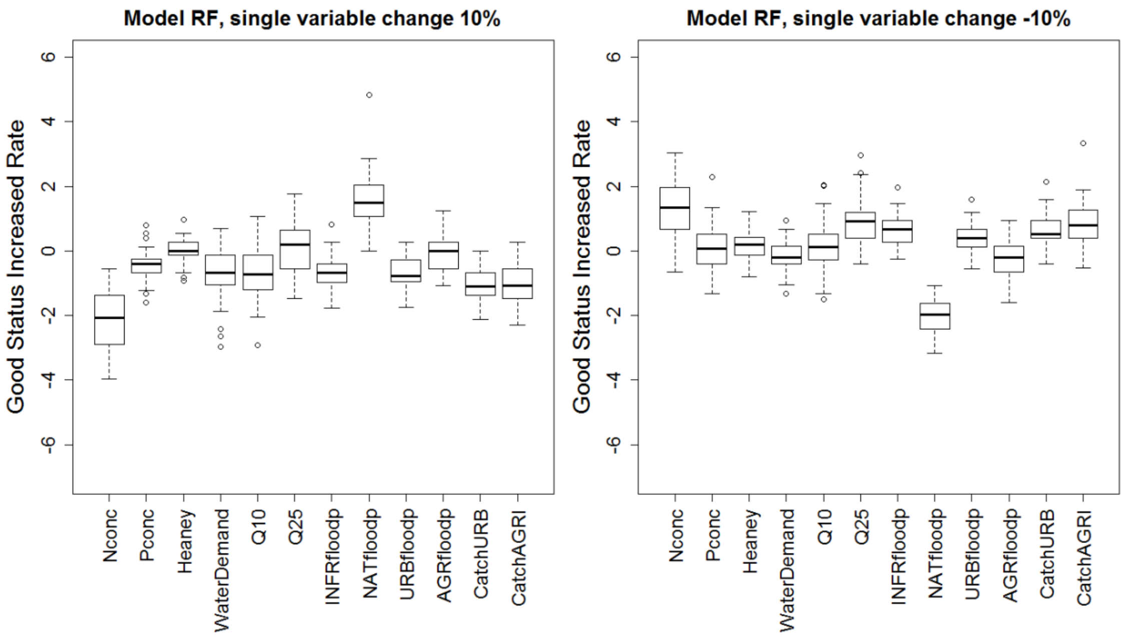

b

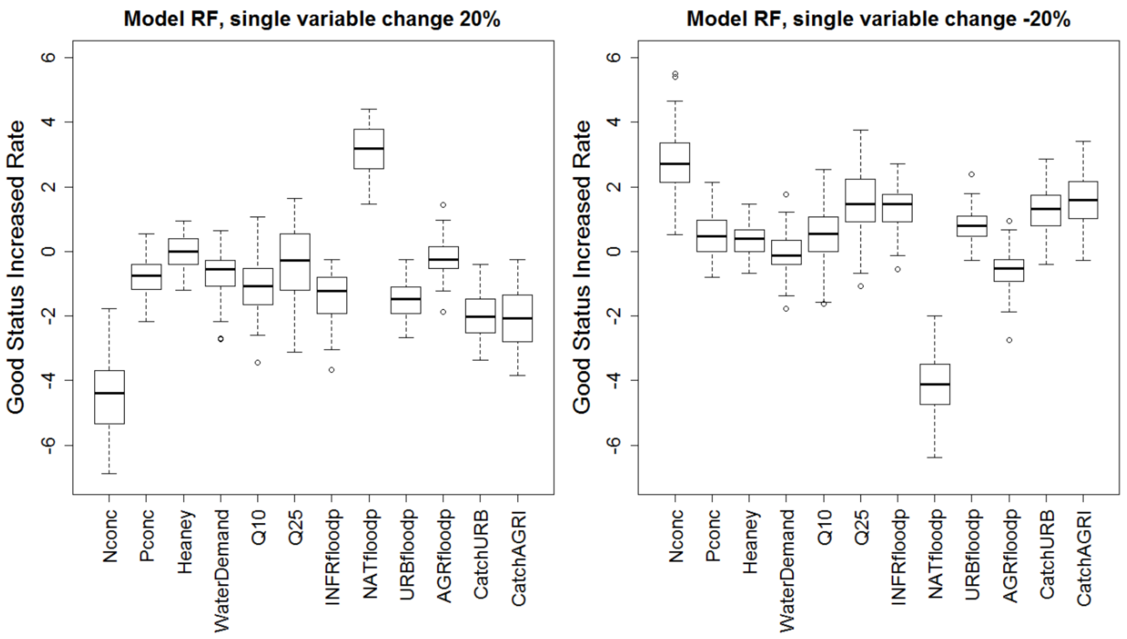

Supplementary Information Figure S4 | Expected effect of changing one pressure at a time on meeting the good ecological status, simulated by the logistic regression (LR) method. a. changing single variable by ± 10%. **b**. changing single variable by ± 20%. The good status increased rate is calculated as the ratio of catchments in less than good ecological status (in the baseline) that under the scenario are predicted to pass to good ecological status.

a

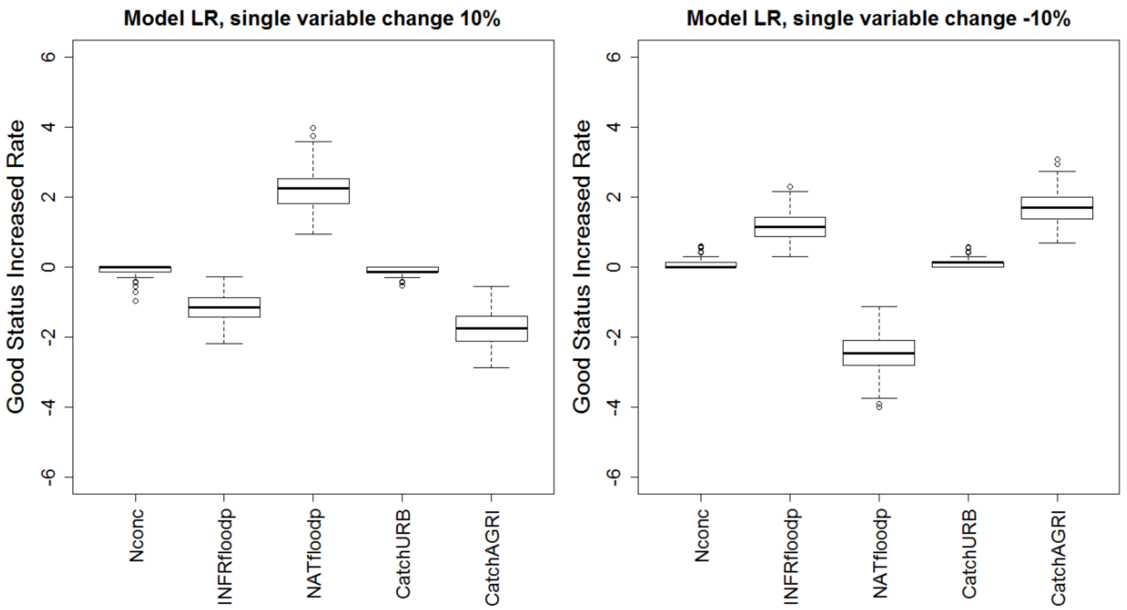

b

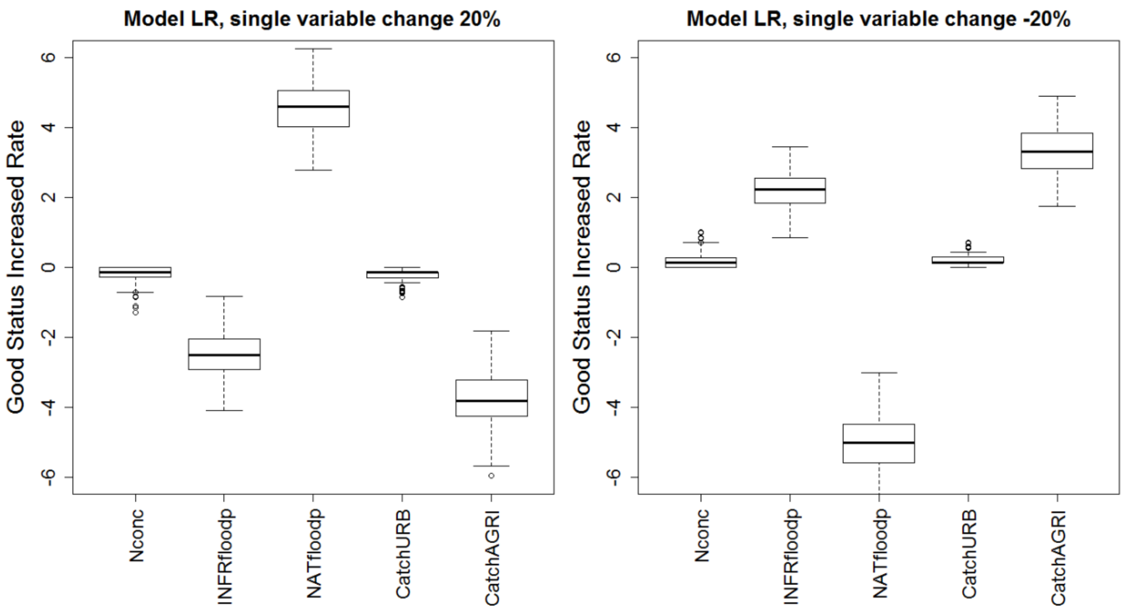

Supplementary Information Figure S5 | Scenarios of further degradation of river ecological status. The scenarios are simulated by the three classification methods: regression tree (RT), logistic regression (LR) and random forest (RF). The scenarios 'further degradation' estimate the effects of contemporary increase of nitrogen concentration in rivers and the decrease of natural areas in floodplains, considering degradation rates of 10% and 20%.

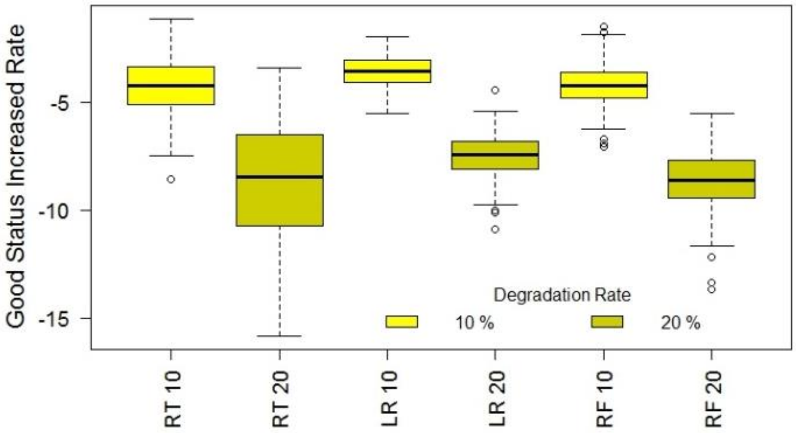

Scenario	Model	Increased rate of good ecological status (*) (median)	Average
Degradation 10%	RT10	-4.3	-4.0
	LR10	-3.6	
	RF10	-4.3	
Degradation 20%	RT20	-8.5	-8.2
	LR20	-7.5	
	RF20	-8.6	

(*) The increased rate is calculated as the ratio of catchments in less than good ecological status (in the baseline) that under the scenario are predicted to pass to good ecological status (see 'Methods').
